# Transcription Factor Antagonism Controls Enteroendocrine Cell Specification from Intestinal Stem Cells

**DOI:** 10.1038/s41598-017-01138-z

**Published:** 2017-04-20

**Authors:** Yumei Li, Zhimin Pang, Huanwei Huang, Chenhui Wang, Tao Cai, Rongwen Xi

**Affiliations:** 1 School of Life Science, Tsinghua University, Beijing, 100084 China; 2 National Institute of Biological Sciences, Zhongguancun Life Science Park 7 Science Park Road, Beijing, 102206 China

## Abstract

The balanced maintenance and differentiation of local stem cells is required for Homeostatic renewal of tissues. In the *Drosophila* midgut, the transcription factor Escargot (Esg) maintains undifferentiated states in intestinal stem cells, whereas the transcription factors Scute (Sc) and Prospero (Pros) promote enteroendocrine cell specification. However, the mechanism through which Esg and Sc/Pros coordinately regulate stem cell differentiation is unknown. Here, by combining chromatin immunoprecipitation analysis with genetic studies, we show that both Esg and Sc bind to a common promoter region of *pros*. Moreover, antagonistic activity between Esg and Sc controls the expression status of Pros in stem cells, thereby, specifying whether stem cells remain undifferentiated or commit to enteroendocrine cell differentiation. Our study therefore reveals transcription factor antagonism between Esg and Sc as a novel mechanism that underlies fate specification from intestinal stem cells in *Drosophila*.

## Introduction

In *Drosophila*, intestinal epithelial homeostasis is maintained by intestinal stem cells (ISCs)^1, 2^. Typically, each ISC divides once, producing one self-renewal cell and one transient progenitor enteroblast (EB) cell. The EB cell then differentiates further into either a mature secretory enteroendocrine (ee) cell or an absorptive enterocyte (EC) cell. Approximately 10–20% of EB cells differentiate into ee cells and 80–90% of EB cells differentiate into EC cells^3^. However the mechanism that controls the differentiation ratio between EC and ee cells remains unknown. It has been reported that the Notch signaling pathway is the main regulator of this cell fate choice. Notch activation promotes EC cell specification, while Notch inactivation induces ee cell specification^1, 2^. Ee cell specification is regulated by proneural genes - *AS/C* genes (*achaete*, *scute*, *lethal of scute*, and *asense*)^4^. The loss of *AS/C* genes inhibits ee cell specification, and overexpression of either Scute (Sc) or Asense (Ase) in ISCs promotes ee cell specification^4^. The protein Slit which is secreted by ee cells can activate Robo receptors that are expressed in ISCs to inhibit ee cell specification, thereby forming a negative feedback mechanism^5^. Recently, Tramtrack (Ttk69), a BTB domain-containing transcriptional repressor, has been reported to regulate ee cell specification^6^. The loss of Ttk69 leads to de-repression of Sc and Ase expression, which subsequently induces the expression of Prospero (Pros), a transcriptional factor that promotes ee cell specification^5–7^.

The transcription factor Escargot (Esg), a homologue of mammalian Slug, encodes a zinc finger motif present in genes of the Snail family of transcription factors^8^. Previous studies in *Drosophila* showed that Esg maintains the diploidy of imaginal cells^9^, regulates cell adhesion and motility in trachea^10^, and acts as a Seizure repressor in a *Drosophila* epilepsy model^11^. Esg can directly interact with Daughterless (Da), thereby preventing Da protein degradation and thus promoting neuronal differentiation^12^. Moreover, studies in the *Drosophila* midgut have established that Esg regulates the maintenance of ISC stemness, controls EC cell specification via repressing the expression of the transcription factor Pdm1, a POU/homeodomain transcription factor, and acts as a regulator of ee cell specification in EB cells by regulating the expression of Amun, a downstream negative regulator of Notch signaling^13, 14^.

The *Drosophila* AS/C-complex, which is composed of four class II HLH proteins, act as transcriptional activators by forming heterodimers with the E-protein Daughterless (Da), a class I HLH protein. AS/C-complex promotes the formation of sensory organs in embryonic and adult peripheral neural systems, and also induces neuroblast formation in the central neural system^15^. The regulation of the *AS/C* genes is complex: they can be induced by the GATA factor Pannier, and can be repressed by the class VI HLH protein Enhancer-of-split (E(spl)) and the class V HLH protein Extramacrochaetae (Emc) during the development of dorsal-central mechanosensory bristles, neurons, and sensory organs^16–19^. Interestingly, an *in vitro* study of cultured S2 cells showed that Sc/Da heterodimer activity can be antagonized by Esg which can bind to the same HLH-family E2 box consensus- binding sequence^9^. However, it is as yet unclear whether this antagonism influences *in vivo* physiology.

Given the similar but opposite roles of Esg and Scute in regulating ee cell specification in the *Drosophila* midgut, we investigated whether Esg and Scute can antagonize each other to regulate ee cell specification. Our genetic results demonstrate that Esg can antagonize Sc activity and thus directly control the expression of Pros which in turn controls ee cell specification.

## Results

### Transiently knocking down *esg* in ISCs promotes ee cell specification

**Figure 1:**
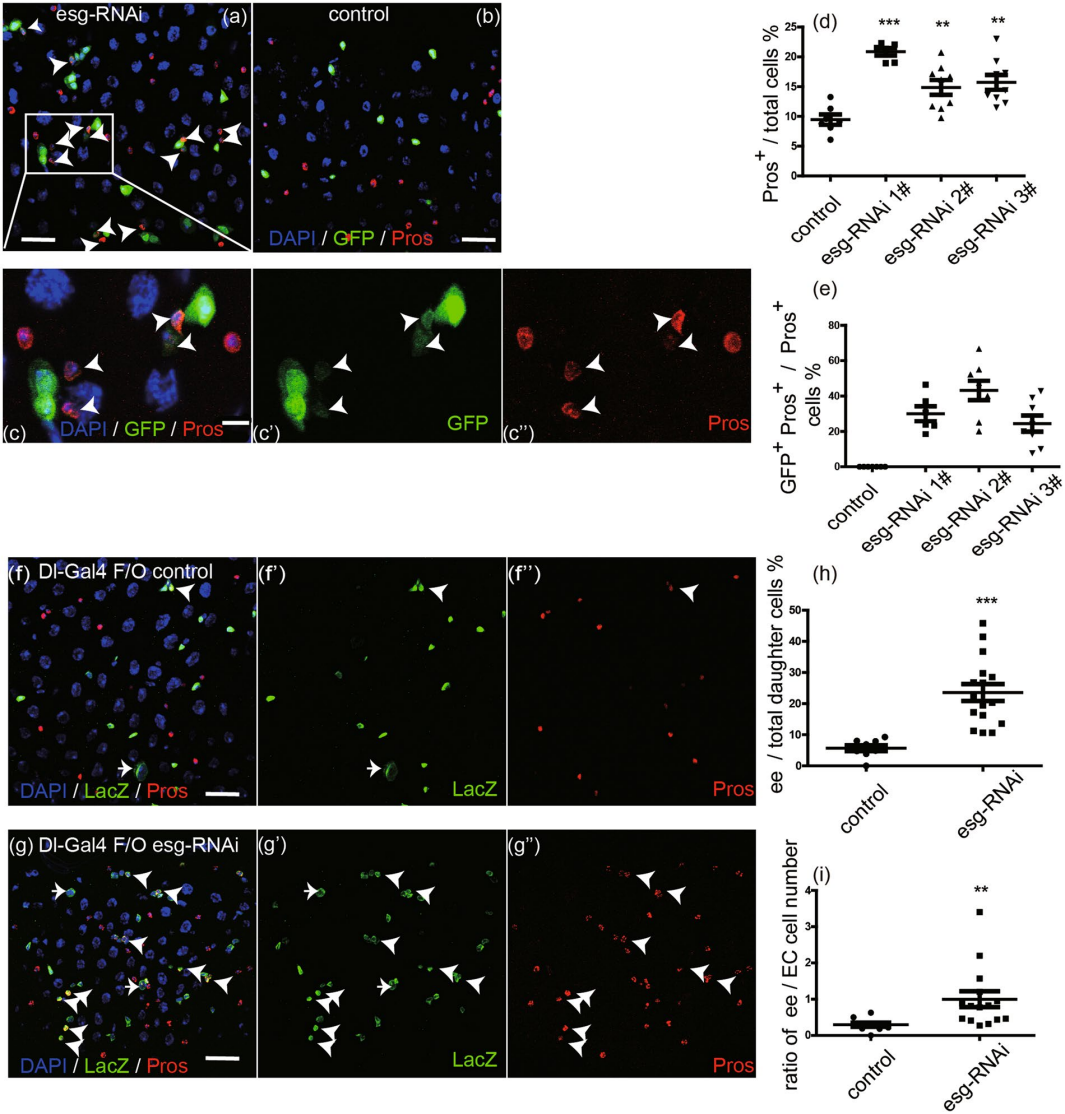
Transiently knocking down *esg* in ISCs promotes ee cell specification. (**a**,**b**) *Esg* knockdown in ISCs induced excess ee cells. Representative images from midguts expressing *UAS-esg-RNAi* or expressing *UAS-GFP* alone (control) 3 days at 29 °C via the *Dl-Gal4* driver. Control image has no GFP^+^ Pros^+^ cells. *Esg-RNAi* image contains more Pros^+^ cells and GFP^+^ Pros^+^ cells (white arrowhead). Samples were stained with DAPI (blue), GFP (green), and Pros (red). Scale bars 20 μm. (**c**) Enlargement of the area outlined in (**a**). Scale bar 5 μm. (**d**) Quantification of Pros^+^ cells in images from control and three independent *esg-RNAi* lines. Each data point is a proportion calculated from an image. (Students’ t test, ***(p < 0.0001), **(p = 0.0046 for 2#, p = 0.0017 for 3#)). (**e**) Quantification of GFP^+^ Pros^+^/Pros^+^ cells in images (**a**,**b**) and another two independent *esg-RNAi* lines. (**f**,**g**) *Esg* knockdown promotes ee cell fate choice. *Dl-Gal4* drives the expression of *UAS-flp* to excise stop codon from the Act < stop < *LacZ* cassette, so the ISC daughter cells show the expression of LacZ. The white arrows point to polyploid EC cells, while the white arrowheads point to Pros^+^ ee cells. Scale bar 20 μm. (**h**) Quantification of Pros^+^ ee/total LacZ^+^ daughter cells in (**f**,**g**). Students’ t test, ***(p < 0.0001). (**i**) Quantification of the ratio of ee/EC daughter cell number in (**f**,**g**). Students’ t test with Welch’s correction, **(p = 0.0067).

To investigate the mechanism through which Esg affects ee cell specification, *UAS-GFP* and *UAS-esg-RNAi* were specifically expressed in ISCs via use of *Dl-Gal4* driver^20^. Because Esg is essential for ISC maintenance, we performed a short-term knockdown experiment and examined the midguts at 3 days after inducing *esg-RNAi* expression, when most ISCs were still maintained. We found small clusters of 3–4 cells that frequently contained Pros^+^ cells (Fig. 1a). Pros status was used to judge ee cell identity. This type of clusters was not frequent in wild-type midgut, in which Pros^+^ cells were dispersed randomly and were fewer in numbers (Fig. 1b). Similar results were obtained with three separate *esg-RNAi* transgenic lines that targeted divergent regions of *esg* (Fig. 1d). These data suggest that knockdown of *esg* promoted ee cell specification. Intriguingly, we also observed that some of the Pros^+^ cells exhibited weak GFP expression (Fig. 1a and c). Similar results were obtained with other two independent *esg*-*RNAi* lines (Fig. 1e). Given that GFP expression is only expected to occur in ISCs in wild-type midgut, our observation of Pros^+^ GFP^+^ cells in *esg* knockdown midgut implies that ee cells are newly generated and still retain some GFP product from mother ISCs. These observations indicate that *esg* knockdown causes ISCs to immediately produce ee cells.

There were no differences in ISC proliferation as directed by Phosphate Histone 3 (PH3, a marker of mitosis) between wild-type midgut and *esg* knockdown midgut, showing that short-term *esg* knockdown did not increase or decrease ISC division (Fig. S1a).

At 7 days after induction of *esg* knockdown, we observed the loss of ISCs (Fig. S1b), a finding consistent with previous reports^13, 14^. Therefore, *esg* knockdown causes ISCs to transiently produce ee cells before ISCs are exhausted.

Next, we used the *Dl-Gal4* lineage system to trace ISC daughter cells to further evaluate the differentiation of ISCs into ee cells. In this system, *UAS-flp* expression was driven by *Dl-Gal4* to excise the stop codons in the *act* < *stop* < *LacZ* cassette to induce LacZ expression. All the daughter cells were thus marked by LacZ expression. In the wild type, the daughter cells differentiated into ee cells and EC cells, or maintained in progenitor cells (Fig. 1f). ISCs with *esg* knockdown had more ee daughter cells (Fig. 1g). Based on our quantification of the ee and EC cell number in all of the daughter cells, we could see that the lineage differentiation into ee cells was obviously increased in *esg* knockdown midgut and also found that the ratio between the ee and EC daughter cells was also increased (Fig. 1h and i). These results are consistent with the previous observations that the knockdown of *esg* in ISCs increases the extent of ee cell specification.

### Knocking down *esg* in EBs does not affect ee cell specification

**Figure 2:**
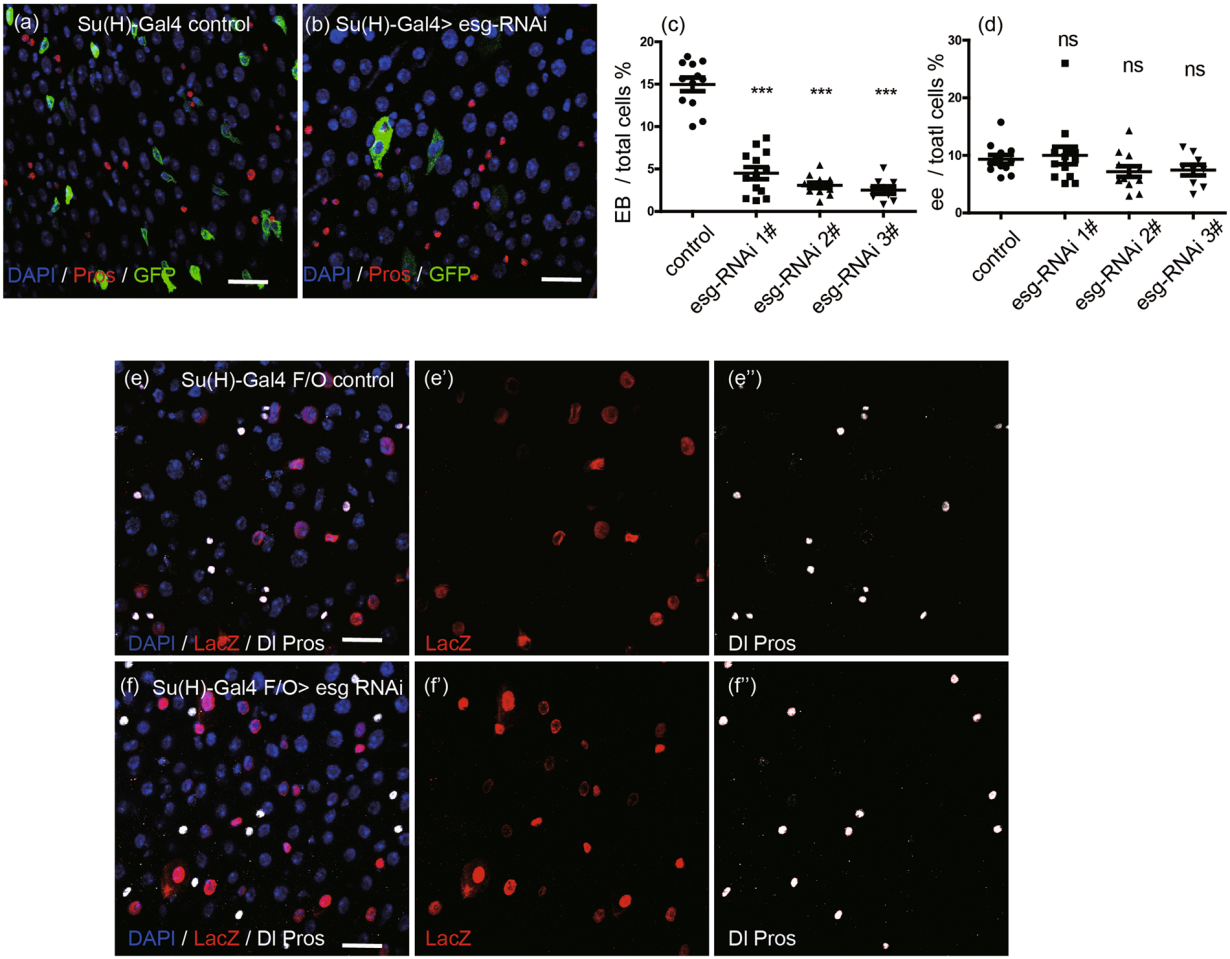
Knocking down *esg* in EBs does not affect ee cell specification. (**a**,**b**) EB cells decrease in *esg-RNAi* image. *UAS-GFP* alone (control) and *UAS-esg-RNAi* were driven by *Su* (*H*)*GBE-Gal4* in EBs at 29 °C, and samples were stained with DAPI (blue), GFP (green), and Pros (red). Scale bars 20 μm. (**c**) Quantification of EB cells in images from control (**a**) and three independent *esg-RNAi* lines. Students’ t test, ***(p < 0.0001). (**d**) Quantification of ee cell number in images from control (a) and three independent *esg-RNAi* lines. (Students’ t test, ns = no significance, 1# (p = 0.7011), 2# (p = 0.0895), 3# (p = 0.1324). (**e**,**f**) Ee cells cannot be traced from EB cells. *Su* (*H*)*-Gal4* drives *UAS-flp* expression to excise the stop codon in the *Act* < *stop* < *LacZ* cassette, inducing LacZ expression in EB lineage cells. Samples were stained with DAPI (blue), LacZ (red), Dl (white circle), and Pros (white nuclei). Scale bars 20 μm.

To further investigate the function of Esg in progenitor EB cells, *esg* knockdown and GFP expression were specifically driven in EB cells via the *Su*(*H*)*GBE-Gal4* driver^20^. Compared to the wile type, transient knockdown of *esg* caused reduction in the number of GFP^+^ EB cells, and the remaining GFP^+^ EB cells showed polyploidy (Fig. 2a and b). The quantification of the distribution of GFP^+^ EB cells in three independent *esg*-*RNAi* lines showed an obvious decrease in the number of EB cells (Fig. 2c). Consistent with the previous report by Korzelius *et al*.^13^, these results suggest that *esg* knockdown in EB cells causes EB cells to rapidly differentiate into EC cells.

Knockdown of *esg* did not obviously affect the population of ee cells, as compared with the wild type as assessed by monitoring Pros expression (Fig. 2a and b). The quantification of the ee cell number in the three independent *esg-RNAi* lines showed no significant differences compared to the wild type (Fig. 2d). We also used the *Su*(*H*)*GBE-Gal4* lineage tracing system, as with our *Dl-Gal4* lineage system experiments introduced above, to trace EB cell fate. In the wild type, all EB cells differentiated into EC cells (Fig. 2e), which was consistent with the results reported by Zeng & Hou, 2015^7^. All of the *esg* knockdown EB cells also differentiated into EC cells (Fig. 2f). These results suggest that *esg* knockdown in EB cells does not affect ee cell specification. The results of our *esg* knockdown and cell-lineage-tracing experiments led us to conclude that *esg* knockdown in EB cells does not alter EB cell fate but rather accelerates the differentiation of EB cells into EC cells.

### Esg and Sc acts in parallel to regulate ee cell specification

In *Drosophila* midgut, the transcription factor Scute (Sc) is specifically expressed in cells with small nuclei, including ISC, EB, and ee cells, and it is known to be required for ee cell specification^4^. Loss of Sc causes deficiency of ee cell differentiation^21^, and ectopic expression of Sc increases ee cell specification^4, 7^.

**Figure 3:**
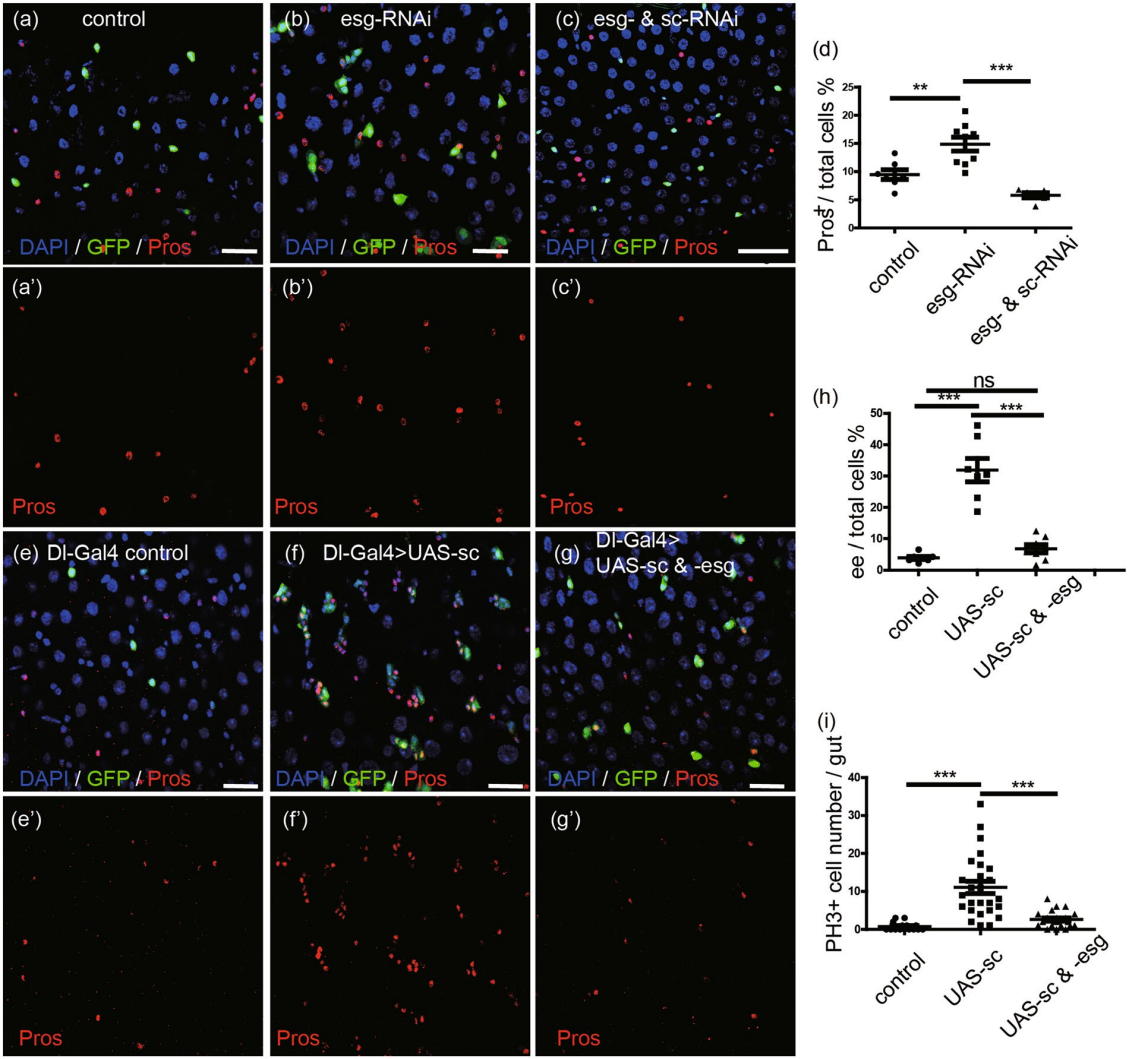
Esg and Sc acts in parallel to regulate ee cell specification. (**a**–**c**) Knocking down *sc* rescues the increase of ee cell number caused by *esg* knockdown. *Dl-Gal4* drives expression of GFP alone (control), *esg-RNAi*, and *esg- & sc-RNAi* at 29 °C for 3 days. Samples were stained with DAPI (blue), GFP (green), and Pros (red). Scale bars 20 μm. (**d**) Quantification of ee cells in images from control (**a**), *esg-RNAi* lines (**b**), and *esg- & sc-RNAi* lines (**c**). Students’ t test, **(p = 0.0046), ***(p < 0.0001). (**e**–**g**) Esg overexpression rescues the clustered ee cell phenotype caused by Sc overexpression. *UAS-GFP* alone (control), *UAS-sc*, and *UAS-sc & -esg* were driven by *Dl-Gal4* at 29 °C for 2 days. Samples were stained with DAPI (blue), GFP (green), and Pros (red). Scale bars 20 μm. (**h**) Quantification of ee cells in images (**e**,**f**,**g**). Students’ t test, ***(p < 0.0001), ns (no significance, p = 0.0711). (**i**) Quantification of PH3^+^ cell number in whole midguts. Students’ t test, ***(p < 0.0001).

Owing to the opposite effects of Esg and Sc in ee cell specification, we examined the genetic relationships between Esg and Sc. *Esg* knockdown was induced for 3 days in ISC cells, which resulted in increased accumulation of ee cells (Fig. 3a,b, quantification in 3d). Simultaneous knockdown of *sc* and *esg* rescued the accumulation of ee cells that was observed in the *esg* knockdown midgut (Fig. 3c, quantification in 3d). Moreover, knockdown of another AS/C complex member, *ase*, also rescued the accumulation of ee cells that was observed in *esg* knockdown midgut (Fig. S2a, quantification in S2b). These results suggest that Sc/Ase acts in parallel or acts downstream of Esg in the regulation of ee cell specification.

Sc overexpression alone was sufficient to induce ee cell specification and to produce ee cell clusters (Fig. 3e,f, quantification in 3h), consistent with previous reports^4^. Overexpression of Esg and Sc together rescued the clustered ee cell phenotype observed with Sc overexpression alone to the random ee cell distribution phenotype in the wild type (Fig. 3g, quantification in 3h). Similar results were observed in the co-overexpression of Sc and Esg clones induced via the *Flp-Out* system (Fig. S2c). We verified that overexpression of Esg could rescue the increased ee cell specification caused by ectopic expression of Sc. Similarly, Esg overexpression could also rescue the ee cell accumulation caused by overexpression of Ase (Fig. S2d,e, quantification in S2f).

PH3 staining revealed that, compared to the wild type, overexpression of Sc induced ISC proliferation, and this effect could be inhibited by overexpression of Esg (Fig. 3i). Those observations indicate that Sc does not simply act downstream of Esg, or vice versa. Instead, Esg and Sc appear to function antagonistically to regulate ee cell specification.

### The repressive effect on Pros by Esg can be antagonized by Sc

**Figure 4:**
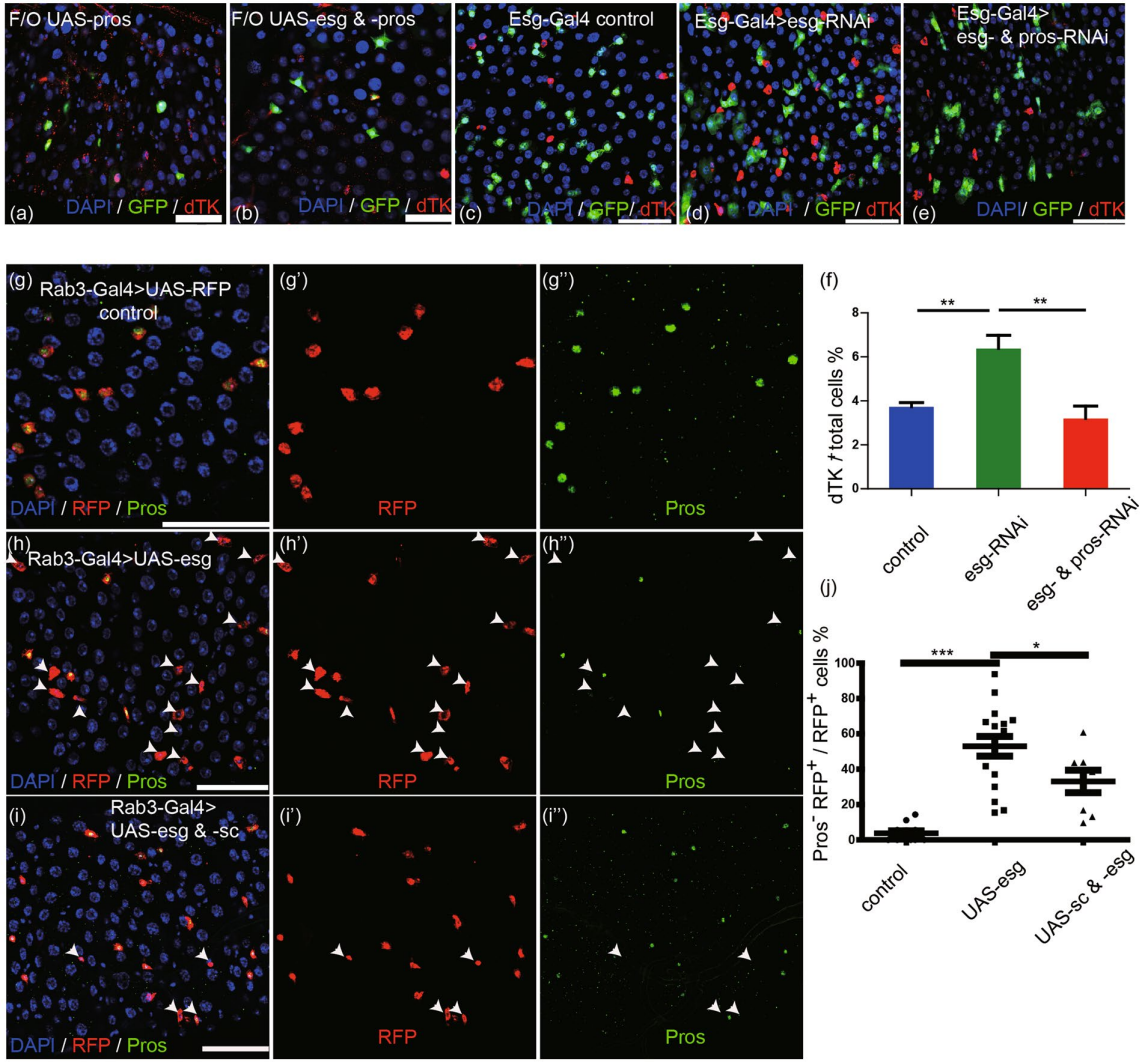
Esg and Sc antagonize to regulate Pros expression. (**a**,**b**) Esg overexpression cannot rescue ISC loss and ee cell specification caused by Pros overexpression. Clones in *UAS-pros* and *UAS-esg & -pros* lines were induced by *Flp-Out* (*F/O*) system, and samples were stained with DAPI (blue) and dTK (red). Scale bars 20 μm. (**c**–**e**) Knocking down *pros* rescues the increase of ee cell number caused by *esg* knockdown. *Esg-Gal4* drives *UAS-GFP* alone (control), *esg-RNAi*, and *esg- & pros-RNAi* at 29 °C for 5 days and samples were stained with DAPI (blue) and dTK (red). Scale bars 50 μm. (**f**) Quantification of dTK^+^ cells in images (**c**,**d**, and **e**). (control, n = 3; *esg-RNAi*, n = 7; *esg-* & *pros-RNAi*, n = 4. Students’ t test with Welch’s correction, **(left, p = 0.0042), **(right, p = 0.0049)). (**g**–**i**) Sc overexpression partially rescues Pros expression that repressed by Esg overexpression. *Rab3-Gal4* drives the expression of *UAS-RFP* alone (control), *UAS-esg*, and *UAS-esg & -sc*. Samples were stained with DAPI (blue) and Pros (green). The white arrowheads point to RFP^+^ Pros^−^ cells. (**j**) Quantification of RFP^+^ Pros^−^/RFP^+^ cells in images (**g**,**h**,**i**). Students’ t test, ***(p < 0.0001), *(p = 0.0417).

The transcription factor Pros is both an ee specific marker and an ee cell fate determination factor. To investigate whether Esg can regulate Pros and thereby alter ee cell specification, we conducted genetics experiments to explore possible regulatory interactions between Esg and Pros. Most Pros overexpression clones induced via *Flp-Out* system contained only one ee cell (Fig. 4a), similar to previous observations^6^. This effect could not be rescued by Esg overexpression (Fig. 4b). However, the increase in ee cell number induced by *esg-RNAi* could be rescued by *pros-RNAi* (Fig. 4c–e, and quantification in 4f). Taken together, these results suggest that Esg acts upstream of Pros to regulate ee cell specification.

Next, we examined whether Esg can regulate Pros expression. Both RFP expression and Esg overexpression were specifically induced in ee cells via the *Rab3-Gal4* driver. Overexpression of Esg resulted in RFP^+^ ee cells that showed reduced or complete loss of Pros expression (Fig. 4h, white arrowheads); this was not observed in wild type, in which Pros expression invariably colocalized with RFP (Fig. 4g). Co-overexpression of Sc and Esg was able to partially rescue this loss of Pros expression phenotype (Fig. 4i). Similar results were obtained via quantification of Pros^−^ RFP^+^/all RFP^+^ cells (Fig. 4j). These results indicate that Sc can antagonize the inhibition activity of Esg on Pros expression. Esg overexpression could not inhibit Pros expression in all of the ee cells. This may be due to variance in *Rab3-Gal4* expression levels in different ee cells. It must also be noted that Sc overexpression could not rescue Pros expression back to the wild type level, possibly because Sc mainly functions as a component of Sc/Da heterodimers^9, 22^, or may relate to the potentially competitive interactions between Esg and Sc in regulating Pros expression.

### Esg and Sc bind to a common promoter region of *pros*

**Figure 5:**
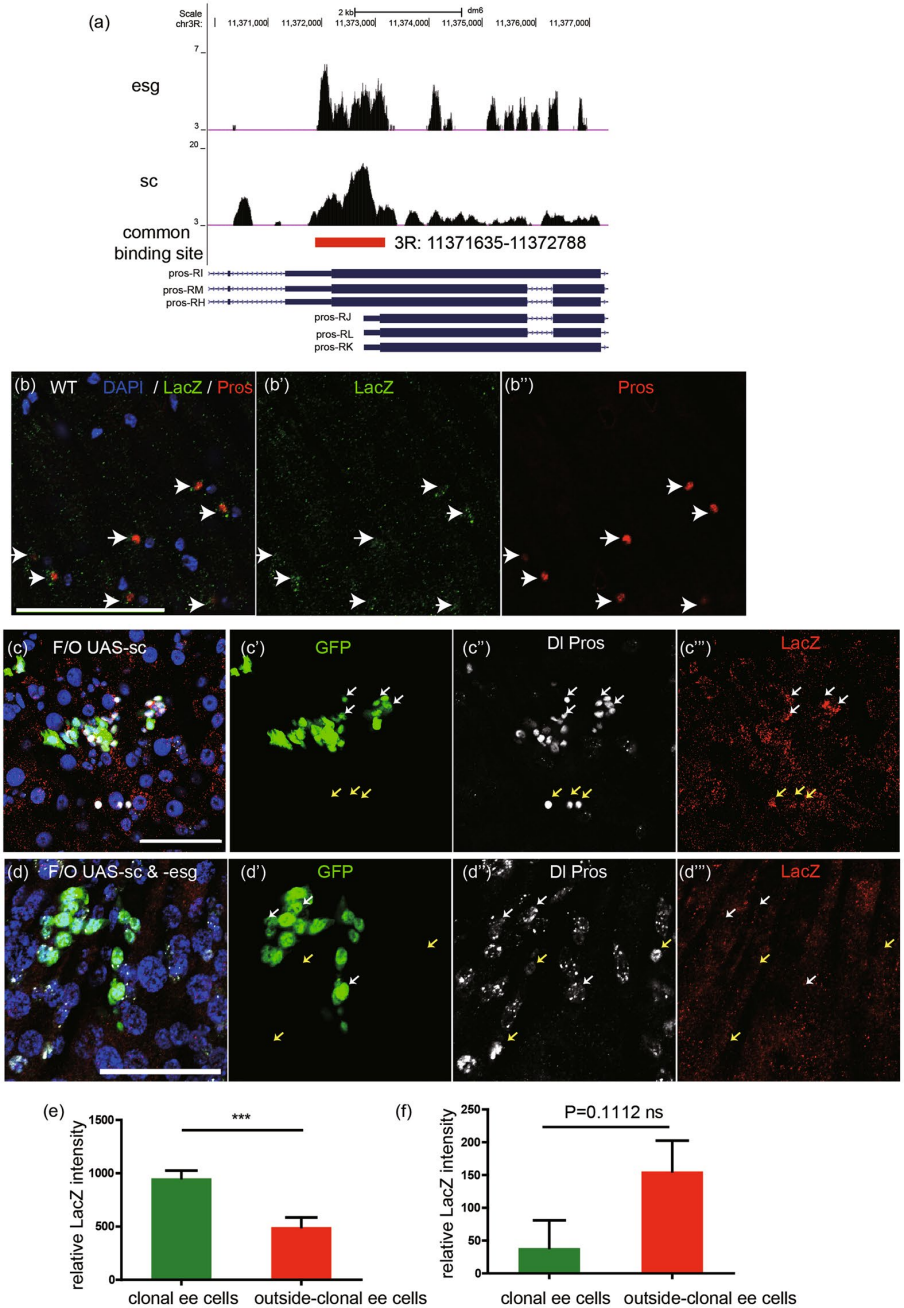
Esg and Sc bind to a common region of *pros* promoter. (**a**) ChIP-seq result analysis of binding sites of Esg and Sc in a *pros* promoter. The upper is peak call of Esg binding sites. The below is peak call of Sc binding sites. The red line directs the common binding site of Esg and Sc in a *pros* promoter. *Pros-RI*, *RM*, *RH*, *RJ*, *RL* and *RK* are different *pros* transcripts. (**b**) This common binding sequence of Esg and Sc drives LacZ expression specifically in ee cells in wild type midgut. Samples were stained with DAPI (blue), LacZ (green), and Pros (red). Scale bar 50 μm. (**c**,**d**) Esg overexpression represses *pros* promoter activation by Sc overexpression. LacZ expression was driven by this common binding sequence. Clonal cells were driven by F/O system, and samples were stained with DAPI (blue), Dl (white circle), Pros (white nuclei), and LacZ (red). White arrows point to clonal ee cells and yellow arrows point to outside-clonal ee cells. Scale bar 50 μm. (**e**) Quantification of relative LacZ intensity between clonal ee cells (n = 23) and outside-clonal ee cells (n = 17) in (**c**). Students’ t test, ***(p < 0.0001). (**f**) Quantification of relative LacZ intensity between clonal ee cells (n = 13) and outside-clonal ee cells (n = 24) in (**d**). (Students’ t test, ns = no significance).

It has been reported that both Esg and Sc can antagonize to bind to E2 box *in vitro* ^9^, however this has not been confirmed *in vivo*. We performed ChIP-seq of Esg and Sc in *Drosophila* adult midguts to investigate this phenomenon. We found that Esg had binding peaks at promoters of *amun* and *nub* (see Methods for details), which were reported to be targets of Esg in midgut^13, 14^. This result indicates that ChIP-seq data is reliable. In light of our ChIP-seq data, six Sc binding peaks and four Esg binding peaks near the *pros* locus were found. Among these binding peaks Esg and Sc showed two common binding peaks. One was located in an exon of *pros*. We thus found only one common binding site (3 R: 11371635–11372788) which located in a *pros* promoter region (Fig. 5a and Table S1). This binding site was about 1.1 kb distant from the 5′UTR of two transcripts *pros*-RL, RJ which had the same size as the reported *pros* transcripts from northern blot analysis in *Drosophila* neuron cells^23, 24^. Following this, to investigate the expression pattern of this common binding site, we generated a LacZ reporter driven by this common binding site. We named this LacZ reporter prosE1-LacZ. We observed that LacZ was specifically expressed in ee cells (Fig. 5b, arrow), implying that this region is the enhancer region of Pros expression in ee cells.

We next used the *Flp-Out* system to examine, *in vivo*, the regulation of *prosE1-LacZ* expression by Sc and Esg. Ectopic expression of Sc resulted in clonal ee cells that showed much higher LacZ expression intensity than ee cells outside the region in which the mosaic clone cells were located (Fig. 5c, quantification in 5e). However, when simultaneously overexpressing Sc and Esg, the clonal ee cells showed similar LacZ expression levels with ee cells located outside of the mosaic clone region (Fig. 5d, quantification in 5f). We thus found that prosE1-LacZ expression can be activated by Sc and can also be inhibited by Esg. Taken together, these results suggest that Esg and Sc may antagonize each other in binding to a common promoter region of *pros* to regulate Pros expression, consequently controlling ee cell specification.

### Esg acts in parallel with Notch while Sc acts downstream of Notch to regulate ee cell specification

Previous studies demonstrated that ee cell specification is mainly regulated by the Notch signaling pathway^1, 2^ and AS/C complex^4^. However, it is still unclear how Notch, Esg, and Sc cooperate to regulate ee cell specification.

**Figure 6:**
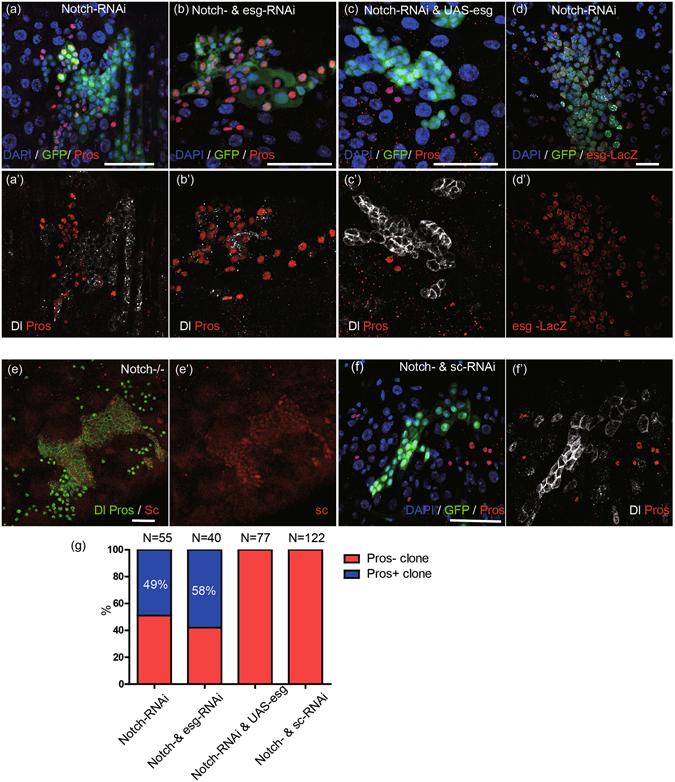
Sc, Esg, and Notch. (**a**,**b**) *Esg* knockdown promotes ee cell specification caused by *Notch* knockdown. *Flp-Out* system was used to induce GFP^+^ clones of expressing *Notch-RNAi*, *Notch- & esg -RNAi*. Samples were stained with DAPI (blue), Dl (white), and Pros (red). Scale bars 20 μm. (**c**) Esg overexpression blocks ee cell specification caused by *Notch* knockdown. *Flp-Out* system was used to induce GFP^+^ clones of expressing *Notch-RNAi & UAS-esg*. Samples were stained with DAPI (blue), Dl (white), and Pros (red). Scale bars 20 μm. (**d**) *Notch* knockdown cannot alter Esg expression level. Esg promoter drives LacZ expression. *Flp-Out* system was used to induce GFP^+^ clones of expressing *Notch-RNAi*, and samples were stained with DAPI (blue) and LacZ (red). Scale bar 20 μm. (**e**) Loss of Notch upregulates Sc expression. MARCM system drives mosaic GFP^+^ clones of *Notch* mutation. Samples were stained with Dl (green, circle), Pros (green, nuclei), and Sc (red). Scale bar 20 μm. (**f**) *Sc* knockdown blocks ee cell specification caused by *Notch* knockdown. *Flp-Out* system was used to induce GFP^+^ clones of expressing *Notch- & sc-RNAi*. Samples were stained with DAPI (blue), Dl (white), and Pros (red). Scale bar 20 μm. (**g**) Quantification of Pros^+^ clones and Pros^−^ clones in midguts from A, B, C, and F. N represents total clone number (Pros^−^ clones (Red) and Pros^+^ clones (blue)).

Notch inactivation generated a similar number of Pros^+^ clones with Pros^+^ cell cluster and Pros^−^ clones with Dl^+^ cell cluster (Figs 6a, S3a, quantification in 6g), which is consistent with previous observations^1–3^. Statistical analysis of the two types of clones showed that co-knockdown of *esg* and *Notch* resulted in more Pros^+^ clones than *Notch* knockdown alone (Fig. 6g), indicating that knockdown of *esg* promoted ee cell specification during Notch inactivation (Fig. 6b). In contrast, ectopic Esg expression completely blocked Pros^+^ clone formation during Notch inactivation (Fig. 6c, quantification in 6g). These results establish that Esg acts either in parallel or acts downstream of Notch to regulate ee cell specification. However, comparing *esg*-*LacZ* reporter expression levels between clonal cells and cells located outside of the mosaic clone, the Notch inactivation did not alter *esg* transcription level (Fig. 6d). Taken together, these results confirm that Esg expression is not regulated by Notch in ISCs and, further, that Esg acts in parallel with Notch to regulate ee cell specification.

Sc expression, which was difficult to detect in cells located outside of Notch^−/−^ clone region, was upregulated in *Notch* mutant clonal cells (Fig. 6e). We found that knockdown of *sc* blocked ee cell formation during Notch inactivation (Fig. 6f, quantification in 6g). This show Sc is required for ee cell formation induced by Notch inactivation. These results imply that Sc acts downstream of Notch to regulate ee cell specification.

## Discussion

**Figure 7:**
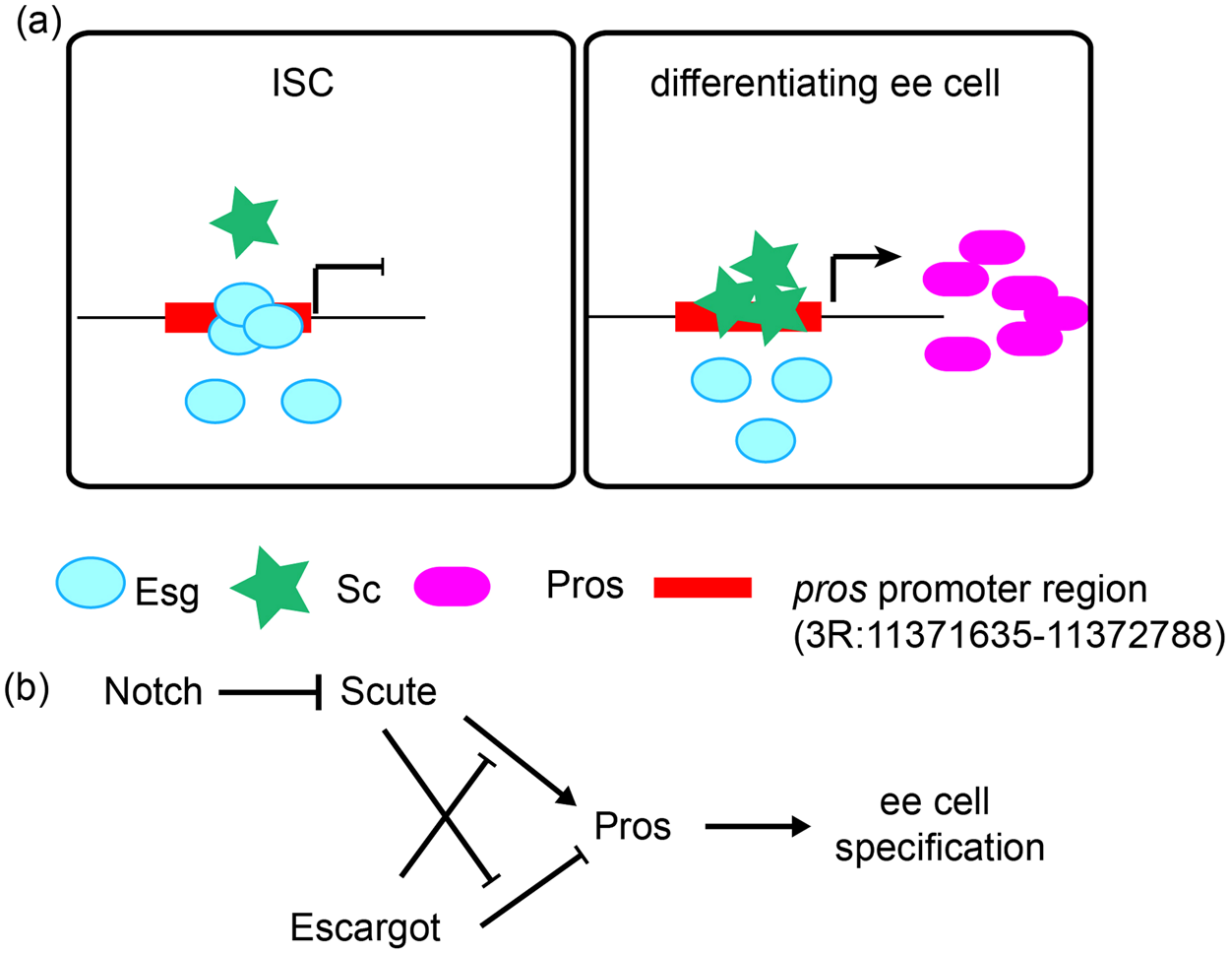
Regulatory model of ee cell specification. (**a**) In ISCs, Esg, which is dominant over Sc, binds to the promoter of *pros*, repressing Pros expression to keep ISC undifferentiated state; in differentiating ee cells, Sc displaces Esg to bind to the promoter of *pros*, thus promoting Pros expression, consequently, ee cell specification. (**b**) Regulatory model of ee cell specification. Sc is upregulated by Notch inactivation to activate Pros expression directly or by antagonizing the repression of Esg on Pros. Esg represses Pros expression directly or by antagonizing the activation of Sc on Pros. Then, the antagonism between Sc and Esg regulates Pros expression to control ee cell specification.

Maintaining tissue homeostasis requires balanced self-renewal and differentiation of tissue-specific stem cells. Here, we demonstrate the antagonism between Snail family transcription factor, Esg, and HLH family transcription factor, Sc, controls ee cell specification from ISCs. Normally, Esg represses Pros, while Sc activates Pros, Pros then induces ee cell specification. Our study suggests that Esg and Sc antagonize each other, likely directly on the *pros* promoter, to control Pros expression, and consequently ee cell specification. In ISCs, Esg is dominant over Sc, Pros expression then remains repressed, and the cells remain undifferentiated. In differentiating ee cells, Notch inactivation upregulated Sc, and then Sc is dominant over Esg, activating Pros expression to promote ee cell specification (Fig. 7a and b). Therefore, our study demonstrates a novel and simple mechanism in which two transcription factors determine the maintenance or differentiation decisions of progenitor cells by antagonistic regulation of a common cell-fate- determination factor.

Our study indicated that *esg* knockdown in ISCs generated many GFP^+^ Pros^+^ cells (Fig. 1a) which appear more frequently than Dl^+^ Pros^+^ cells observed in wild type midguts^7, 25^. And Su(H)GBE^+^ EBs after *esg* knockdown could not generate ee cells, suggesting that the ee cell specification after *esg* knockdown is not via EBs. These results support that ee cells were differentiated from distinct precursors as shown in previous reports^7, 25^. Zeng and Hou, 2015 suggested that Dl^+^ PH3^+^ Pros^+^ cells differentiated into ee cells through symmetric or asymmetric division^7^. And Guo and Ohlstein, 2015 showed that ee cells were generated through symmetric or asymmetric localization of Pros, and overexpression of Ase induced asymmetric Pros localization in all division ISCs^25^. However, whether *esg* knockdown could change the ratio between symmetric and asymmetric division of ISCs and whether *esg* knockdown could induce Pros asymmetric localization need more study.

The up-regulation of Sc was observed in almost all Dl^+^ cells in the *Notch* ^−/−^ clone. And Sc upregulation promotes ee cell specification^4^. However, how those Dl^+^ cells with up-regulated Sc expression could not differentiate into ee cells is not clear. One explanation could be that, in the cells of *Notch* ^−/−^ clonal region, unchanged expression of Esg inhibited ee cell specification. Therefore, Esg down-regulation is necessary for ee cell specification. However, what controls down-regulation of Esg and whether or not the down-regulation of Esg is the primary reason for this remains unclear.

Ectopic expression of Esg could completely prevent ee cell formation observed in Notch inactivation (Fig. 6c) but not in Sc overexpression (Fig. 3g). Maybe owing to Sc expression level was lower in Notch inactivation than in Sc overexpression, Esg had the competitive advantage of binding *pros* promoter, repressing Pros expression. These results suggest that the expression level of Esg and Sc need to be kept in a precise ratio to control Pros expression to balance cell fate specification, although so far we did not know what the ratio it would be.

Snail family members have been reported, *in vitro*, to antagonize the transcriptional activation of HLH family members through the competitive binding to E2 box CANNTG^26–29^. In *C*. *elegans*, Snail family member Ces-1 and Hlh3/Hlh2 heterodimer (similar to *Drosophila* Da/Sc) binds to an E box in regulatory region of Egl-1, an activator of cell death, to regulate Egl-1 activation, thereby controlling cell death^28^. In mouse, Snail family members Snai 1 and Snai 2 can compete with HLH family member Ascl2 to bind to E2 box, regulating expression of tumor repressor Epbh3 to control tumor metastasis^29^. These observations suggest that the antagonistic mechanism between Snail family and HLH family controls a variety of cellular events and are evolutionarily conserved. We propose that the antagonistic function between Snail and HLH family members in controlling stem cell differentiation could be evolutionarily conserved as well.

## Methods

### Stocks and plasmid construction

The following stocks were used in this study: UAS*-esg-RNAi:* 1#, TRiP HMS02538 and 3# TRiP JF03134 (Tsinghua Fly Center)*;* 2# (v9793, VDRC)*;* UAS*-sc-RNAi*: 1# TRiP JF02104 (THU2205, Tsinghua Fly Center) and 2# TRiP GL01130 (THU4591, Tsinghua Fly Center); *UAS-esg-3HA and UAS-sc-3HA* (FlyORF); *Su*(*H*)*GBE-Gal4* and *Dl-Gal4* (gift from Xiankun Zeng and Steven Hou, National Cancer Institute, USA); *esg-LacZ* (#10359, BDSC); *UAS-Notch-RNAi*, *rab3*-*Gal4*, *tub-Gal80* ^*ts*^, *UAS-Flp*, *Act* < *stop* < *LacZ*, *UAS-esg* and *UAS-sc* were all obtained from BDSC.

3R:11371635–11372788 was amplified from the DNA extracted from wild-type flies, and was then cloned into the C4PLacZ plasmid using the *KpnI* and *NotI* sites.

### Lineage tracing system

Crosses were made at 18 °C to obtain the following genotypes: *UAS*-*Flp tub*-*Gal80* ^*ts*^/*esg*-*RNAi*; *Dl*-*Gal4*/*Act* < *stop* < *LacZ*, *UAS-Flp tub-Gal80* ^*ts*^/+; *Dl-Gal4*/*Act* < *stop* < *LacZ*, *Su(H)GBE-Gal4UAS-GFP*/*esg-RNAi*; *Act* < *stop* < *LacZ*, *tub-Gal80* ^*ts*^/*UAS-Flp*, *Su(H)GBE-Gal4 UAS-GFP*/+; *Act* < *stop* < *LacZ tub-Gal80* ^*ts*^/*UAS-Flp*, *Su(H)GBE-Gal4 UAS-GFP*/*UAS-Flp*; *Act* < *stop* < *LacZ tub-gal80* ^*ts*^/*esg-RNAi*. Following eclosion, these genotype flies were treated at 29 °C for 3–7 days.

### Mosaic analysis

The MARCM^30^ and Flp-Out techniques^31^ were used to generate mosaic homozygotic clones in intestines. Mosaic clones were induced in 3 to 5-day-old females via heat shock in a water bath once for 1 h at 37 °C. Flies were subsequently fed with regular food and were supplied with yeast paste. Flies were transferred every 2 days prior to dissection and analysis.

### Immunohistochemistry and microscopy

Drosophila adult midguts were dissected in PBS and fixed for 30 min at room temperature in a mixture of 4% formaldehyde and n-heptane^32^. The following primary antibodies were used: mouse anti-Dl (DSHB, 1:100); mouse anti-Pros (DSHB, 1:300); rabbit anti-β-galactosidase (Cappel, 1:6000); mouse anti-PH3 (Cell Signaling), rabbit anti-Tachykinin (a gift from Dick Nassel, Stockholm University, Sweden; 1:3000); Rat anti-Sc (1:300, a gift from Steve Crews); rabbit anti-Pros (1:200, a gift from Yuh-Nung Jan). Pictures were captured with a Zeiss Meta or a Nikon A1-R confocal microscope, Data were processed using LSM Image Browser or NIS, and were adjusted using Photoshop.

### LacZ intensity analysis

NIS software was used to measure LacZ expression intensity in cells and to assess background intensity levels in the pictures captured with the Nikon A1-R. Background intensity values were calculated as the average value of the intensity of seven random areas. The LacZ intensity values were calculated as the difference between cell signal intensity and background intensity.

### CHIP-seq

*Esg-Gal4*, *Tub-gal80* ^*ts*^ was crossed with *UAS-esg-3HA* and *uas-sc-3HA* at 18 °C. 3–5 day old progenies were shifted to 29 °C to induce overexpression (7 days for Esg and 3 days for Sc). Whole midgut was dissected on ice in a vessel containing dissection buffer (PBS + 0.1 mg/ml PMSF + 1x prote*ase* inhibitor (Roche)). Dissected intestine tissues were then fixed for 15 min at 37 °C in a solution composed of 200 µl of the dissection buffer and 5.5 µl of 37% formaldehyde (pre-warmed to 37 °C in a water bath for 1 min)^33^. 2000–3000 intestines were prepared thusly to generate enough material for the CHIP-seq analysis. Cells were lysed with a homogenizer, and samples were sonicated to yield DNA fragments of 200–700 bp in size as input DNA. Final ChIP DNA was obtained using anti-HA antibody (Abcam) incubated and an additional DNA purification step, all as described in the manual for the ChIP-IT® High Sensitivity kit (Active Motif). Both the ChIP DNA and input DNA were sequenced (BIOPIC High-sequencing Center, Peking University).

The ChIP–seq data were mapped to the Drosophila melanogaster reference genome (BDGP6) using the Bowtie software package (version 1.1.2), with three mismatches allowed. Uniq mapping reads were permitted in the next analysis. We used the MACS software package (version 1.4.2) to call significantly-enriched peaks with a qvalue cutoff of 0.05 relative to the input control samples. To generate the bigwig files, we normalized the tag counts in each bin according to the total number of reads. Input reads were processed in the same way, and their normalized signal intensity values were subtracted from the ChIP-Seq tracks. More details are available in NCBI GEO data series GSE84283.

(Weblink: https://www.ncbi.nlm.nih.gov/geo/query/acc.cgi?token=mvwtqqggtxobbuf&acc=GSE84283).

## Electronic supplementary material

supplementary information

## References

[1] Micchelli CA, Perrimon N (2006). Evidence that stem cells reside in the adult Drosophila midgut epithelium. Nature.

[2] Ohlstein B, Spradling A (2006). The adult Drosophila posterior midgut is maintained by pluripotent stem cells. Nature.

[3] Ohlstein B, Spradling A (2007). Multipotent Drosophila intestinal stem cells specify daughter cell fates by differential notch signaling. Science.

[4] Bardin AJ, Perdigoto CN, Southall TD, Brand AH, Schweisguth F (2010). Transcriptional control of stem cell maintenance in the Drosophila intestine. Development.

[5] Biteau B, Jasper H (2014). Slit/Robo signaling regulates cell fate decisions in the intestinal stem cell lineage of Drosophila. Cell reports.

[6] Wang C, Guo X, Dou K, Chen H, Xi R (2015). Ttk69 acts as a master repressor of enteroendocrine cell specification in Drosophila intestinal stem cell lineages. Development.

[7] Zeng X, Hou SX (2015). Enteroendocrine cells are generated from stem cells through a distinct progenitor in the adult Drosophila posterior midgut. Development.

[8] Whiteley M, Noguchi PD, Sensabaugh SM, Odenwald WF, Kassis JA (1992). The Drosophila gene escargot encodes a zinc finger motif found in snail-related genes. Mechanisms of development.

[9] Fuse N, Hirose S, Hayashi S (1994). Diploidy of Drosophila imaginal cells is maintained by a transcriptional repressor encoded by escargot. Genes & development.

[10] Tanaka-Matakatsu M, Uemura T, Oda H, Takeichi M, Hayashi S (1996). Cadherin-mediated cell adhesion and cell motility in Drosophila trachea regulated by the transcription factor Escargot. Development.

[11] Hekmat-Scafe DS, Dang KN, Tanouye MA (2005). Seizure suppression by gain-of-function escargot mutations. Genetics.

[12] Yang DJ (2010). Slug, mammalian homologue gene of Drosophila escargot, promotes neuronal-differentiation through suppression of HEB/daughterless. Cell Cycle.

[13] Korzelius J (2014). Escargot maintains stemness and suppresses differentiation in Drosophila intestinal stem cells. The EMBO journal.

[14] Loza-Coll MA, Southall TD, Sandall SL, Brand AH, Jones DL (2014). Regulation of Drosophila intestinal stem cell maintenance and differentiation by the transcription factor Escargot. The EMBO journal.

[15] Garcia-Bellido A, de Celis JF (2009). The complex tale of the achaete-scute complex: a paradigmatic case in the analysis of gene organization and function during development. Genetics.

[16] Garcia-Garcia MJ, Ramain P, Simpson P, Modolell J (1999). Different contributions of pannier and wingless to the patterning of the dorsal mesothorax of Drosophila. Development.

[17] Jimenez G, Ish-Horowicz D (1997). A chimeric enhancer-of-split transcriptional activator drives neural development and achaete-scute expression. Molecular and cellular biology.

[18] Skeath JB, Carroll SB (1994). The achaete-scute complex: generation of cellular pattern and fate within the Drosophila nervous system. FASEB journal: official publication of the Federation of American Societies for Experimental Biology.

[19] Van Doren M, Ellis HM, Posakony JW (1991). The Drosophila extramacrochaetae protein antagonizes sequence-specific DNA binding by daughterless/achaete-scute protein complexes. Development.

[20] Zeng X, Chauhan C, Hou SX (2010). Characterization of midgut stem cell- and enteroblast-specific Gal4 lines in drosophila. Genesis.

[21] Amcheslavsky A (2014). Enteroendocrine cells support intestinal stem-cell-mediated homeostasis in Drosophila. Cell reports.

[22] Murre C (1989). Interactions between heterologous helix-loop-helix proteins generate complexes that bind specifically to a common DNA sequence. Cell.

[23] Matsuzaki F, Koizumi K, Hama C, Yoshioka T, Nabeshima Y (1992). Cloning of the Drosophila prospero gene and its expression in ganglion mother cells. Biochemical and biophysical research communications.

[24] Vaessin H (1991). prospero is expressed in neuronal precursors and encodes a nuclear protein that is involved in the control of axonal outgrowth in Drosophila. Cell.

[25] Guo, Z. & Ohlstein, B. Stem cell regulation. Bidirectional Notch signaling regulates Drosophila intestinal stem cell multipotency. *Science* **350**, doi:10.1126/science.aab0988 (2015). 10.1126/science.aab0988 PMC5431284 26586765

[26] Kataoka H (2000). A novel snail-related transcription factor Smuc regulates basic helix-loop-helix transcription factor activities via specific E-box motifs. Nucleic acids research.

[27] Nakakura EK (2001). Mammalian Scratch: a neural-specific Snail family transcriptional repressor. Proceedings of the National Academy of Sciences of the United States of America.

[28] Thellmann M, Hatzold J, Conradt B (2003). The Snail-like CES-1 protein of C. elegans can block the expression of the BH3-only cell-death activator gene egl-1 by antagonizing the function of bHLH proteins. Development.

[29] Ronsch K (2015). SNAIL1 combines competitive displacement of ASCL2 and epigenetic mechanisms to rapidly silence the EPHB3 tumor suppressor in colorectal cancer. Molecular oncology.

[30] Lee T, Luo L (1999). Mosaic analysis with a repressible cell marker for studies of gene function in neuronal morphogenesis. Neuron.

[31] Struhl G, Basler K (1993). Organizing activity of wingless protein in Drosophila. Cell.

[32] Lin G, Xu N, Xi R (2008). Paracrine Wingless signalling controls self-renewal of Drosophila intestinal stem cells. Nature.

[33] Tran V, Gan Q, Chen X (2012). Chromatin Immunoprecipitation (ChIP) using Drosophila tissue. Journal of visualized experiments: JoVE.

