# Supplementary material for: Transcription Factor Antagonism Controls Enteroendocrine Cell Specification from Intestinal Stem Cells
Source: Sci Rep. 2017 Apr 20;7:988. doi: 10.1038/s41598-017-01138-z (PMC5430544; doi:10.1038/s41598-017-01138-z)
Supplement: Supplementary file 1 — supplementary information [file 41598_2017_1138_MOESM1_ESM.pdf]

Transcription Factor Antagonism Controls Enteroendocrine Cell Specification from Intestinal Stem Cells

Yumei Li[1,2*], Zhimin Pang[2], Huanwei Huang[2], Chenhui Wang[2], Tao Cai[2], Rongwen Xi[2*]

[1]School of Life Science, Tsinghua University, Beijing 100084, China.

[2]National Institute of Biological Sciences, Zhongguancun Life Science Park 7 Science Park Road, Beijing 102206, China.

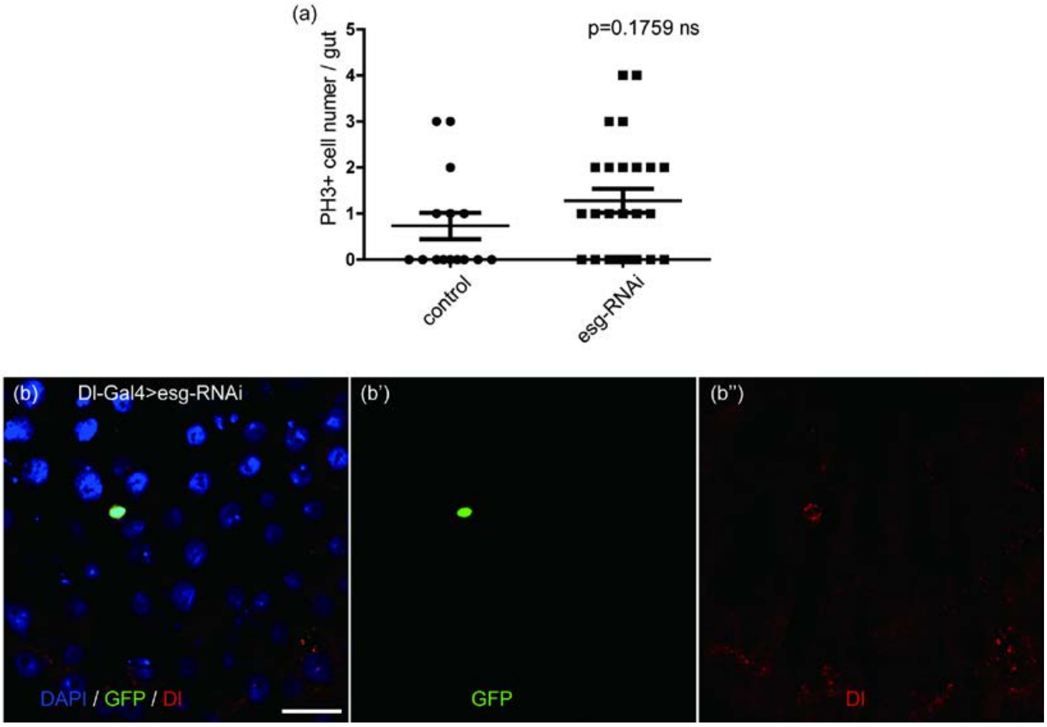

Supplementary Figure S1. *Esg* knockdown causes ISC loss.

(a) Quantification of PH3+ cell number of whole midgut in control and *esg-RNAi* driven by *Dl-Gal4*. (Students' t test, ns= no significance). (b) *Esg* knockdown in ISCs causes ISC loss. *UAS-GFP* and *UAS-esg-RNAi* were driven by *Dl-Gal4* in ISCs at 29°C for 7 days. Samples were stained with DAPI (blue), GFP (green), and Dl (red). Scale bars 20μm.

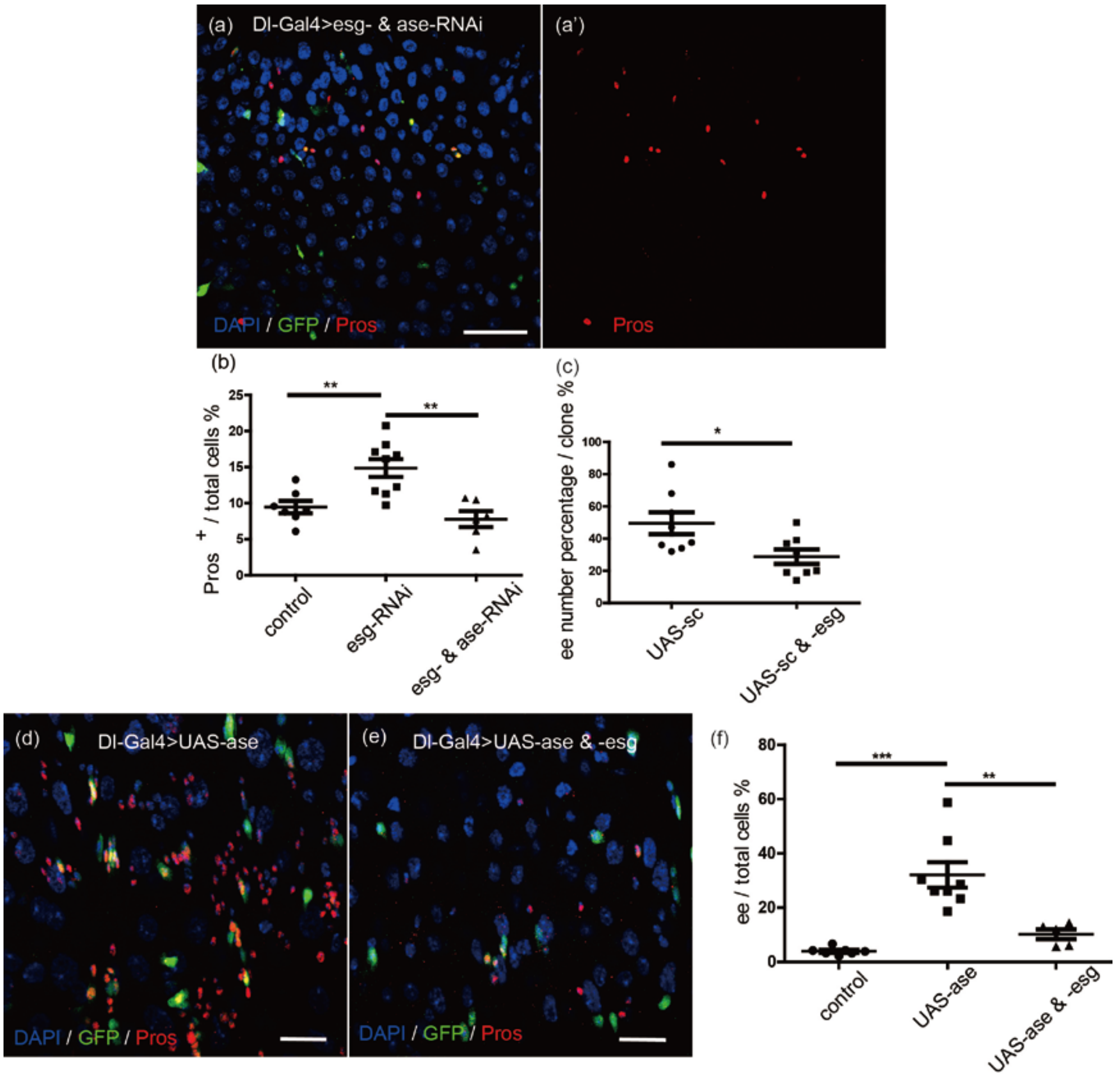

Supplementary Figure S2. Esg acts in parallel with Ase to regulate ee cell specification.

(a) Knocking down *ase* rescues the increase of ee cell number caused by *esg* knockdown. *Dl-Gal4* drives expression of *esg- & ase-RNAi* at 29°C. Samples were stained with DAPI (blue), GFP (green)， and Pros (red). Scale bars 20μm. (b) Quantification of ee cells in images from control (Fig. 3a), *esg-RNAi* lines (Fig. 3b), and *esg- & ase-RNAi* (a). Students' t test, ** (P=0.0046, 0.0015). (c) Quantification of ee / clonal cells induced via *Flp-Out* system. Students' t test, *(p=0.0229). (d, e) Esg overexpression rescues increased ee cell specification caused by Ase overexpression.

UAS-ase and *UAS-esg* were driven by *Dl-Gal4*, and samples were stained with DAPI (blue), GFP (green), and Pros (red). Scale bars 20μm. (f) Quantification of ee / total cells from images of d, e induced by *Dl-Gal4*. Students't test, *** (p<0.0001), ** (p=0.0045).

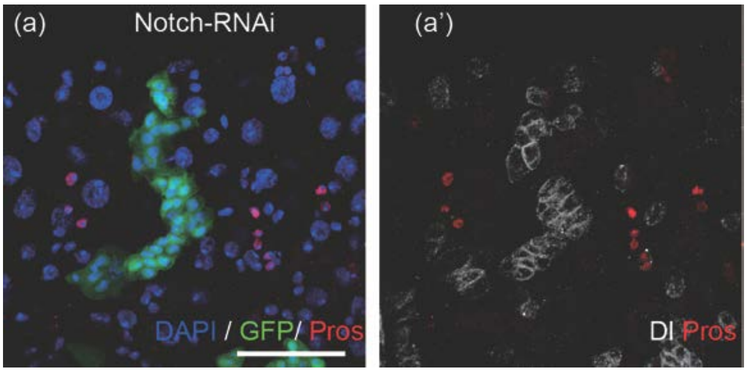

Supplementary Figure S3. Knocking down *Notch* via *Flp-Out* system generates Pros$^-$ clones only with Dl$^+$ cells.

(a). Samples were stained with DAPI (blue), GFP (green), Dl (white), and Pros (red). Scale bars 20μm.

Supplementary Table S1. Two common binding regions of Esg and Sc are located close to *pros*.

Six binding regions of Sc and four binding regions of Esg are located close to the gene *pros*. Among these regions, two common binding regions of Esg and Sc are found.

Supplementary Table S1. Analysis of Esg and Sc binding regions close to *pros* by ChIP-seq

Sc-ChIPSeq

chr	start	end	length	-10* LOG10 (pvalue)	fold_ enrichment	FDR(%)	Annotation	Distance to TSS	Nearest PromoterID	Gene Name
3R	11365947	11366902	956	396.5	11.43	4.1	intron (FBtr0304608, intron 2 of 7)	-6363	FBtr0304605	pros
3R	11367060	11367671	612	231.84	9.26	3.22	intron (FBtr0304608, intron 2 of 7)	-5422	FBtr0304605	pros
3R	11367819	11370001	2183	665.44	13.29	5.71	intron (FBtr0304608, intron 2 of 7)	-3878	FBtr0304605	pros
3R	11370239	11370846	608	141.72	6.29	3.98	intron (FBtr0304608, intron 3 of 7)	-2245	FBtr0304605	pros
3R	11371616	11377286	5671	1676.93	10.09	25	exon (FBtr0304607, exon 1 of 5)	1663	FBtr0304607	pros
3R	11377566	11379163	1598	959.27	13.15	10.14	intron (FBtr0304608, intron 6 of 7)	5577	FBtr0304607	pros

Esg-ChIPSeq

chr	start	end	length	-10* LOG10 (pvalue)	fold_ enrichment	FDR(%)	Annotation	Distance to TSS	Nearest PromoterID	Gene Name
3R	11327577	11328792	1216	81.04	3.62	2.88	promoter-TSS (FBtr0304604)	-295	FBtr0304604	pros
3R	11371635	11373445	1811	182.17	4.04	6.73	promoter-TSS (FBtr0304607)	-248	FBtr0304605	pros
3R	11375006	11376118	1113	83.8	3.41	3.11	exon (FBtr0304607, exon 1 of 5)	2774	FBtr0304606	pros
3R	11388431	11389435	1005	79.99	3.84	2.78	intron (FBtr0304603, intron 6 of 7)	16145	FBtr0304606	pros

common binding regions of Esg and Sc

chr	start	end	length	-10* LOG10 (pvalue)	fold_ enrichment	FDR(%)	Annotation	Distance to TSS	Nearest PromoterID	Gene Name
3R	11371635	11373445	1811	182.17	4.04	6.73	promoter-TSS (FBtr0304607)	-248	FBtr0304605	pros
3R	11375006	11376118	1113	83.8	3.41	3.11	exon (FBtr0304607, exon 1 of 5)	2774	FBtr0304607	pros
